# Frequencies of CYP2C9 polymorphisms in North Indian population and their association with drug levels in children on phenytoin monotherapy

**DOI:** 10.1186/s12887-016-0603-0

**Published:** 2016-05-14

**Authors:** Nagendra Chaudhary, Madhulika Kabra, Sheffali Gulati, Yogendra Kumar Gupta, Ravindra Mohan Pandey, Bal Dev Bhatia

**Affiliations:** Department of Pediatrics, All India Institute of Medical Sciences, New Delhi, India; Department of Pediatrics, Universal College of Medical Sciences, Bhairahawa, Nepal; Genetic Unit, Department of Pediatrics, All India Institute of Medical Sciences, New Delhi, India; Division of Pediatric Neurology, Department of Pediatrics, All India Institute of Medical Sciences, New Delhi, India; Department of Pharmacology, All India Institute of Medical Sciences, New Delhi, India; Department of Biostatistics, All India Institute of Medical Sciences, New Delhi, India

**Keywords:** Epilepsy, Neurocysticercosis, CYP2C9 polymorphism, Phenytoin monotherapy

## Abstract

**Background:**

Phenytoin, mainly metabolized by cytochrome P450 enzyme system, has a narrow therapeutic index and may have adverse effects due to inter-individual variation in the dose requirement and genetic polymorphisms. This cross-sectional study was done to study the prevalence of cytochrome P450 CYP2C9 polymorphisms in Indian epileptic children and to see the effect of polymorphisms on serum levels in epileptic children on phenytoin monotherapy.

**Methods:**

We studied 89 epileptic children of North Indian population, randomly selected, to see the genotypic and allelic frequency of CYP2C9 and its association with drug levels on phenytoin monotherapy. Analysis was done using STATA 9 Software. The results were analyzed as prevalence at 95 % C.I. (Confidence Interval). The difference in mean phenytoin serum levels between wild and mutant alleles was tested using Student`s T test for independent samples. P value less than 0.05 was considered statistically significant.

**Results:**

CYP2C9*1, *2 & *3 allelic frequencies were 85.4, 4.5 and 10.1 % respectively. CYP2C9*3 allelic group showed significantly higher serum phenytoin levels compared to the wild variants (*P* = 0.009). There was no statistically significant difference in the dose received (*P* = 0.12) and side effects of CYP2C9*2 and CYP2C9*3 genotypes (*P* = 0.442 and 0.597 respectively) when compared with wild variant.

**Conclusion:**

CYP2C9*3 is more common than *2 in the present study. All the polymorphisms demonstrated in our study were heterozygous with no homozygosity. Serum phenytoin levels are higher in polymorphic groups (*3) which suggest their poor metabolizing nature. Genotyping may help to avoid toxicity and concentration-dependent adverse effects.

**Electronic supplementary material:**

The online version of this article (doi:10.1186/s12887-016-0603-0) contains supplementary material, which is available to authorized users.

## Background

Epilepsy is a common disorder in pediatric practice, with a prevalence of 5.59 per 1000 population with no gender or geographical differences in Indian population [1]. Phenytoin is one of the commonly prescribed drugs in children for seizure control. CYP2C9 may be involved in 80–90 % of metabolism of phenytoin and therefore polymorphisms in CYP2C9 may result in significant reduction in the metabolism of phenytoin and can enhance clinical toxicity of the drug [2–4]. CYP2C9 gene is located on the long arm of chromosome 10 at 10q24, has 10 exons and 1847 bases coding DNA which codes for 490 amino acid protein. CYP2C9*2 polymorphism results due to c.430C > T nucleotide change resulting in p.Arg144Cys amino acid change (rs#1799853) whereas CYP2C9*3 results due to change in c.1075A > C resulting in p.Ile359leu amino acid change (rs#1057910). CYP2C9*1 allelic groups have normal enzyme activity whereas the CYP2C9*2 (Cys 144) allelic groups have reduced enzyme activity, and even lower activity in CYP2C9*3 (Leu359) variants. In Caucasians, the most common variant of CYP2C9 is CYP2C9*2 (10–13 % of the population), whereas the frequency of CYP2C9*3 varies from 5–9 % [5–7]. In Asians and Africans, these two alleles appear at lower frequency than seen in the Caucasians [8]. In Chinese and Japanese, the CYP2C9*2 allele has not been detected [9–11]. Previous genotyping reports in different ethnic groups in India demonstrate wide differences in the distribution of the CYP2C9 alleles.

## Methods

### Baseline characteristics

**Fig. 1:**
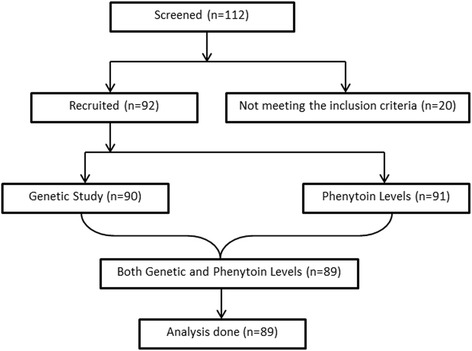
Work plan for the study

This is a cross-sectional observational study done between June 2010 to May 2012, where 89 epileptic children (Males:55, Females: 34) of North Indian origin between age groups 5–12 years with mean age 9.29 (±2.7) years on phenytoin were finally included for analysis (Fig. 1). Sample size was calculated by anticipating 50 % prevalence of cytochrome P450 CYP2C9 in epilepsy patients to fall within 10 % points of the true proportion with 95 % confidence [12]. Cases were enrolled from Out-patient department and Neurocysticercosis Clinic of All India Institute of Medical Sciences. Majority (93.3 %) of cases included were of neurocysticercosis while rest were having Idiopathic epilepsy (6.7 %). These patients were on phenytoin monotherapy for at least 1 month or more [Mean duration: 12.7 months, Median (min, max): 7 (1–78) months] and were on same drug regimen at the time of drug level measurement. Doses were adjusted once the phenytoin level was >20 mcg/ml. None of the children under study had hepatic or renal dysfunction. Children on polytherapy (more than one antiepileptic drugs), those with genetic syndromes and on drugs that interfere with the metabolism of phenytoin were excluded. Informed written consent was obtained from parents. The study was approved by the Institute`s ethics committee (Office of ethics subcommittee, All India Institute of Medical Sciences, Room no 102, 1^st^ floor, Old OT block, Ansari Nagar, New Delhi-110029, Ref No: IESC/T-182/2010).

### DNA extraction and genotyping

CYP2C9*2 which codes for amino acid change Arg144Cys was detected by PCR-RFLP as described [13] using the following primers; [Forward primer: 5′-cac tgg ctg aaa gag cta aca gag-3′ (24 bases) and Reverse primer: 5′-gtg ata tgg agt agg gtc acc cac-3′ (24 bases)], to amplify a 375 bp amplicon in a 25 μL PCR mix comprising 2.5 μL 10X buffer, 2.5 μL of 2 mM dNTPs, 1 μL of 10 μM each forward and reverse primers, 0.25 μL Taq polymerase, 100.5 μL of sterile water and 1 μL of 100 ng/μL genomic DNA. PCR methodology has been explained in Additional file 1.

**Fig. 2:**
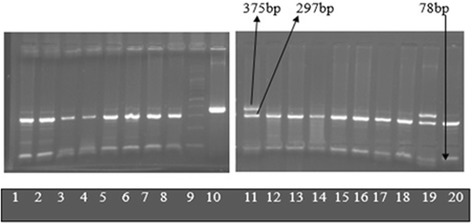
Electrophoresis on 2 % agarose gel after digestion with Ava II (Abbreviations: bp-base pairs). Lanes 1–8, 12–18, 20: *1/*2. Lanes 11 and 19: *1/*2. Lane 9: 100 bp ladder. Lane 10: Unrestricted PCR product

Unrestricted PCR products were of 375 bp. After restriction digestion, homozygote TT samples at c.430 gave 375 bp only (similar to unrestricted PCR products) whereas heterozygotes (CT samples) gave 375 bp, 297 bp and 78 bp products. Homozygote CC samples gave 297 bp and 78 bp products only (Fig. 2).

**Fig. 3:**
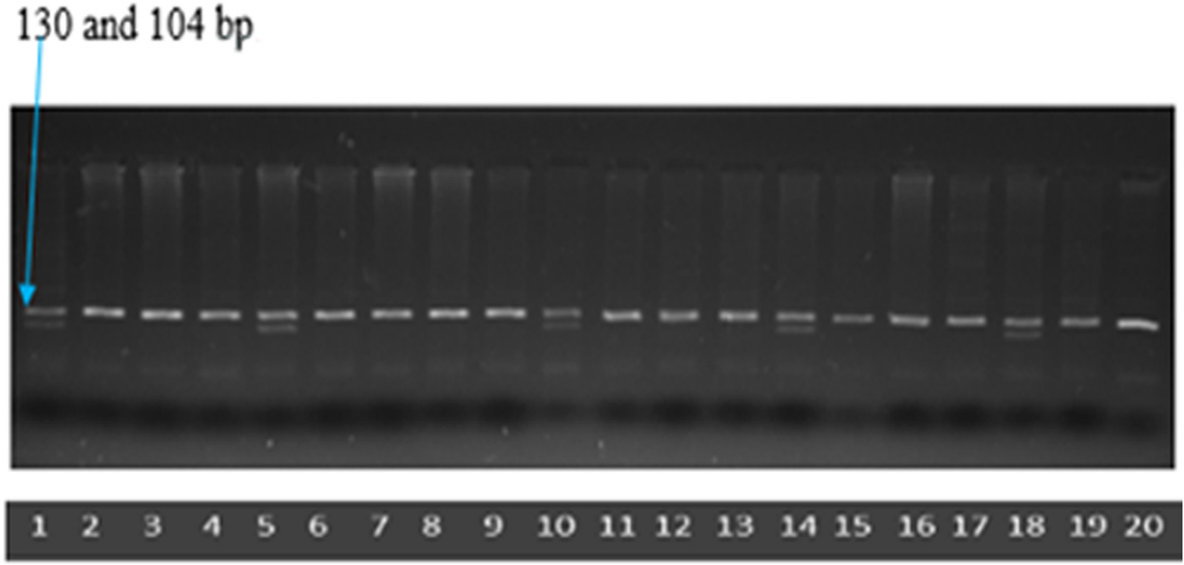
Electrophoresis on 3 % agarose gel after digestion with Sty I (Abbreviations: bp- base pairs). Lanes 1, 5, 10, 14, 18: *1/*3. Lanes 2–4, 6–9, 11–13, 15–17 and 19: *1/*1. Lane 20: Unrestricted PCR product

CYP2C9*3 which is responsible for amino acid change Ile359Leu was detected by PCR-RFLP assay [13] using following primers: Forward primer: 5′-agg aag aga ttg aac gtg tga-3′ (21 bases) and Reverse primer: 5′-ggc agg ctg gtg ggg aga agg cca a-3′ (25 bases). PCR conditions were the same as described (Additional file 1). Unrestricted PCR products were of 130 bp. After restriction digestion, homozygote AA samples at c.1075 gave 130 bp only (similar to unrestricted PCR products) whereas heterozygotes (AC samples) gave 130 bp, 104 bp and 26 bp products. Homozygote CC samples gave 104 bp and 26 bp products only (Fig. 3).

### Phenytoin estimation

2–3 ml blood sample was collected in plain vial the next morning after giving the evening dose of phenytoin. The sample was centrifuged immediately at 10,000 RPM for 10 min. The separated serum was then transferred to micro-centrifuge tubes and stored at −80 °C till phenytoin levels was estimated. Phenytoin level in serum was estimated by High Performance Liquid Chromatography (HPLC). Shimadzu (Prominence) HPLC equipment with Photo Diode Array Detector (PDA) was used at 210 nm. Agilent Zorbax SB-C18 column was used. Flow rate was at 1.2 ml/min. The mobile phase consisted of following ingredients: Potassium dihydrogen orthophosphate buffer (50 mM): Acetonitrile: Methanol 58:21:21 (v/v/v). The mobile phase was filtered through 0.22 μm membrane filter and degassed prior to use. 200 μl of serum was then vortexed mixed with 20 μl diazepam (3 mg/ml) as internal standard, then again it was vortexed and mixed with 200 μl acetonitrile (HPLC grade) for 1 min. The mixture was centrifuged at 15000 rpm for 12 min and the supernatant was separated. 25 μl of the supernatant was filtered through a 0.4 μM filter and was directly injected into the HPLC system. Patient samples were estimated for phenytoin levels by using a standard calibration curve.

### Statistical analysis

Genotype frequencies in the study population were checked for Hardy-Weinberg equilibrium. Analysis was done using STATA 9 Software. The results were analyzed as prevalence at 95 % C.I. (confidence Interval). The difference in mean phenytoin serum levels between wild and mutant alleles was tested using Student’s *T* test for independent samples. P value less than 0.05 was considered statistically significant.

## Results

The frequency of CYP2C9*1/*2/*3 alleles were 85.4, 4.5 and 10.1 % respectively. The frequency for *1/*1, *1/*2, *1/*3 and *2/*3 genotypes was 71.9, 7.9 , 19.1 % and 1.1 % respectively (Table 1). A single individual was heterozygous for both *2 and *3 polymorphism. CYP2C9*2 genotyping showed 91 % to be wild (CC) and 9 % were heterozygous (CT). No homozygous polymorphism was identified in this study population as expected from the Hardy Weinberg equilibrium. The frequency of wild alleles (C) was 95.5 % and the mutant allele (T) was 4.5 % (Table 2). Genotyping for CYP2C9*3 showed prevalence of 79.8 % for the wild (AA) and 20.2 % for the mutant group (AC). Allele distribution was 89.9 % for the wild and 10.1 % for the mutant. All of the polymorphisms detected were heterozygous. No homozygosity was identified (Table 3). The means for phenytoin doses required in the CC and CT groups did not vary significantly, neither the difference in phenytoin levels in these two groups was significant (Table 4). The mean doses (mg/kg) in AA and AC groups were 5.3 and 4.8 with no statistically significant results but the median phenytoin levels compared in both the groups were statistically significant (*P* = 0.009) (Table 5). The frequency of side effects (gum hypertrophy, ataxia, hirsuitism) in both the polymorphic groups was not statistically significant (*P* = 0.442 and 0.597 for *2 and *3 genotypes) (Table 6).

**Table 1:** CYP2C9 alleles and genotypes

CYP2C9 allele and genotype	CYP2C9 allele frequencies (*n* = 89)	95 % Confidence interval (%)
CYP2C9*1	85.4 %	80.21–90.59
CYP2C9*2	4.5 %	1.45–7.55
CYP2C9*3	10.1 %	5.67–14.53
CYP2C9*1/*1	71.9 %	62.56–81.24
CYP2C9*1/*2	7.9 %	2.27–13.45
CYP2C9*1/*3	19.1 %	10.93–27.27
CYP2C9*2/*3	1.1 %	0–3.27
CYP2C9*2/*2	0 %	0
CYP2C9*3/*3	0 %	0

**Table 2:** Genotypes and alleles of CYP2C9*2 with prevalence and 95 % CI

CYP2C9*2 genotypes	Number (*n* = 89)	Prevalence (%)	95 % CI
CC	81	91	83–96
CT	8	9	3–16
Alleles	No of Alleles	Frequency	95 % CI
C	170	95.5	91–98
T	8	4.5	1.9–8

**Table 3:** Genotypes and alleles of CYP2C9*3 with prevalence and 95 % CI

CYP2C9*3 genotypes	Number (*n* = 89)	Prevalence (%)	95 % CI
AA	71	79.8	69–87
AC	18	20.2	12–30
Alleles	No of Alleles	Frequency	95 % CI
A	160	89.9	84–94
C	18	10.1	6–15

**Table 4:** Comparing wild and mutant genotypes of CYP2C9*2 for dose (mg/kg) and drug level (mcg/ml)

Variables	CC (*n* = 81)	CT (*n* = 8)	*P* value
Dose (mean ± SD)	5.2 ± 1.15	5.1 ± 0.95	0.89
Phenytoin level^a^ (median)	6.8	9.5	0.74

^a^Non parametric test applied as variability was high (Wilcoxon rank-sum test)

**Table 5:** Comparing wild and mutant genotypes of CYP2C9*3 for dose (mg/kg) and drug level (mcg/ml)

Variables	AA (*n* = 71)	AC (*n* = 18)	*P* value
Dose (mean ± SD)	5.3 ± 1.20	4.8 ± 0.69	0.12
Phenytoin level^a^ (Median)	5.9	18.8	0.009

^a^Non parametric test applied as variability was high (Wilcoxon rank-sum test)

**Table 6:** Percentage of side effects in *2 and *3 groups

	Side effects		*P* value	Gum hypertrophy	Hirsuitism	Ataxia
	Yes	No				
CYP2C9*2 genotypes						
CC	5 (5.6 %)	76 (85.4 %)	0.442	4 (4.5 %)	0	1 (1.1 %)
CT	1 (1.1 %)	7 (7.9 %)		1 (1.1 %)	1 (1.1 %)	0
CYP2C9*3 genotypes						
AA	4 (4.5 %)	67 (75.3 %)	0.597	4 (4.5 %)	1 (1.1 %)	0
AC	2 (2.2 %)	16 (18.0 %)		1 (1.1 %)	0	1 (1.1 %)

## Discussion

The frequency of *2 alleles in our study was 0.045 which is less than the reported in Italian, Greek, Russian, Swedish and Iranian Population. Frequency of *3 in our study population was higher than in Chinese, Japanese, Americans, Russian, and Swedish (Table 7). CYP2C9*2 genotypes are negligible or almost absent in the Chinese and Japanese [8–10] while Caucasians have higher frequencies of *2 as compared to *3 genotypes [6, 9, 14]. CYP2C9*3 alleles in the Italian and our population was almost similar. The frequencies of CYP2C9 genotypes are given in Table 5.

**Table 7:** Comparison of CYP2C9 (*1, *2, *3) in different population in relation to the North Indian population

Ethnicity	Number	*1	*2	*3
Chinese Han [9]	196	0.974	0	0.026
African American [9]	200	0.985	0.010	0.005
European American [9]	200	0.860	0.080	0.060
Japanese [10]	436	0.979	0	0.021
Sweden [26]	430	0.819	0.107	0.074
Egypt [7]	247	0.817	0.118	0.062
Russian [6]	290	0.827	0.105	0.067
Italian [27]	360	0.777	0.125	0.097
UK [14]	297	0.841	0.106	0.052
Japan [8]	828	0.976	0	0.023
Greek [5]	283	0.79	0.128	0.081
Iran [28]	200	0.873	0.127	0
Italian [29]	150	0.648	0.253	0.098
Chinese Mongolian [11]	560	0.970	0	0.030
Indian Studies				
Ethnicity	Number	*1	*2	*3
Tamilian population [15]	135	0.9	0.03	0.07
South Indian population [16]	346	0.88	0.04	0.08
Northern Indian population [17]	102	0.95	0.049	0.039
Present study in North Indian population (Delhi)	89	0.85	0.045	0.10

The frequencies of CYP2C9*2 in Indian population have found to be between 3–5 % whereas CYP2C9*3 ranges from 4–8 % [15–17]. The allele frequencies of *2 and *3 in Indian population published till date were found to be almost similar to the present study. A study done in North Indian population by Rathore et al. revealed similar *2 allelic frequencies with lower *3 allelic frequencies (0.10 Vs 0.039). Most of these studies have been carried out on smaller sample size. Our study also had a small sample size. Therefore, multi-centric studies with large sample size should be carried out to solve these discrepancies of the allelic frequency in different studies in the same population.

We also studied association of the CYP2C9*2 and *3 polymorphisms with phenytoin levels. The median phenytoin levels (mcg/ml) in the *2 genotypes, both wild (CC) and mutant (CT) was 6.8 and 9.5 respectively (not significant). We generally use phenytoin at the dose of 5–8 mg/kg/day and maintain a drug level between 10–20 mcg/ml at our center. Statistically significant difference in the drug levels between the CYP2C9*3 wild and mutant genotypes (*P* = 0.009) was observed in our study suggesting the poor metabolizing nature in polymorphic groups; although there was no significant toxicity in those groups.

Recent pharmacogenetic studies have demonstrated the importance of polymorphism with phenytoin levels [13, 18–20]. In a recent study by Vander Weide et al. demonstrated that patients with at least one mutant CYP2C9 allele required a lower dose of phenytoin to achieve a therapeutic serum drug concentration than did the patients with two normal alleles [21]. Lee at al also demonstrated that CYP2C9*3 variants in Korean patients had higher propensity to develop phenytoin induced cutaneous adverse reactions [22]. Kesavan et al. also demonstrated a higher phenytoin levels and toxicity in CYP2C9*2 and CYP2C9*3 allelic variants [23].

Similarly the mean serum concentration of phenytoin of the polymorphic patients with epilepsy was higher than that for the wild-type alleles both in the monotherapy and polytherapy patients in a study done by Ozkaynakci A et al. [24].

In a recent study done by Yamamoto Y et al. concluded that genotyping could help in estimating the optimum target dose of phenytoin and may contribute to avoid toxicity and concentration-dependent adverse effects [25]. These results show the importance of the genetic polymorphism analysis of the main metabolizing enzyme groups of phenytoin for the dose adjustment.

## Conclusions

In summary, both the *2 and *3 allelic variants are commonly seen in the Indian population with CYP2C9*3 being more common than *2 in the present study. All the polymorphisms demonstrated in our study were heterozygous. No homozygosity was seen in our study which suggests that the homozygous polymorphism is rare in this population. The frequency of polymorphism has been found to be different in different population in India itself and some studies in the same population has shown conflicting results which can be solved by conducting larger multi-centric studies. Although *3 group had significantly higher serum phenytoin level when compared to *1 group, they did not have significantly higher toxicity. As the number of patients with toxicity in our study was small, no further conclusive recommendations could be made for clinical implications. Genotyping of the CYP2C9 gene in patients on antiepileptic drugs (eg. Phenytoin) may help to overcome the drug toxicity, choose the right molecule and guide in therapeutic drug monitoring.

### Ethics

The study was approved by the Institute’s ethics committee (Office of ethics subcommittee, All India Institute of Medical Sciences, Room no 102, 1^st^ floor, Old OT block, Ansari Nagar, New Delhi-110029, Ref No: IESC/T-182/2010).

### Consent to participate

Informed and written consent was taken to participate in the study.

### Consent to publish

Consent to publish was obtained from the parents.

### Availability of data and materials

Data has been provided in the materials and methods section of the manuscript. If required, individual data can be obtained from Genetic unit, Department of Pediatrics, All India institute of medical Sciences, New Delhi, India.

## Additional file

Additional file 1: Polymerase chain reaction. PCR was performed by initial denaturation at 94 °C for 2 min followed by 35 cycles of denaturation at 94 °C for 30 s, annealing at 60 °C for 10 s and extension at 72 °C for 1 min followed by final extension at 72 °C for 7 min. The products were run at 250 V in a horizontal electrophoresis system (Bangalore Genie) on a 2 % agarose gel to check for amplification. 5 μL of PCR products (375 bp) were digested overnight at 37 °C with *Ava II* (New England Biolabs) for CYP2C9*2 genotyping. A 130 bp amplicon was digested by Sty 1 (*Eco 1301*) (Fermentas International Inc) for CYP2C9*3 genotyping. After the overnight digestion, the digested DNA was run along with the undigested PCR product on a 2 % agarose gel with ethidium bromide at 150 V in a horizontal electrophoresis system and visualized under UV light. (PDF 82 kb)

## Abbreviations

bp: base pair
CI: confidence interval
CYP: cytochrome P 450
HPLC: high performance liquid chromatography
mcg: microgram
ml: milliliters
PCR: polymerase chain reaction
RFLP: restriction fragment length polymorphism

## References

[1] Sridharan R, Murthy BN (1999). Prevalence and pattern of epilepsy in India. Epilepsia.

[2] Goldstein JA (2001). Clinical relevance of genetic polymorphisms in the human CYP2C subfamily. Br J Clin Pharmacol.

[3] Takanashi K, Tainaka H, Kobayashi K, Yasumori T, Hosakawa M, Chiba K (2000). CYP2C9 Ile359 and Leu359 variants: enzyme kinetic study with seven substrates. Pharmacogenetics.

[4] Rettie AE, Haining RL, Bajpai M, Levy RH (1999). A common genetic basis for idiosyncratic toxicity of warfarin and phenytoin. Epilepsy Res.

[5] Arvanitidis K, Ragia G, Iordanidou M, Kyriaki S, Xanthi A, Tavridou A (2007). Genetic polymorphisms of drug-metabolizing enzymes CYP2D6, CYP2C9, CYP2C19 and CYP3A5 in the Greek population. Fundam Clin Pharmacol.

[6] Gaikovitch EA, Cascorbi I, Mrozikiewicz PM, Brockmöller J, Frötschl R, Köpke K (2003). Polymorphisms of drug-metabolizing enzymes CYP2C9, CYP2C19, CYP2D6, CYP1A1, NAT2 and of P-glycoprotein in a Russian population. Eur J Clin Pharmacol.

[7] Hamdy SI, Hiratsuka M, Narahara K, El-Enany M, Moursi N, Ahmed MS-E (2002). Allele and genotype frequencies of polymorphic cytochromes P450 (CYP2C9, CYP2C19, CYP2E1) and dihydropyrimidine dehydrogenase (DPYD) in the Egyptian population. Br J Clin Pharmacol.

[8] Mushiroda T, Ohnishi Y, Saito S, Takahashi A, Kikuchi Y, Saito S (2006). Association of VKORC1 and CYP2C9 polymorphisms with warfarin dose requirements in Japanese patients. J Hum Genet.

[9] Sullivan-Klose TH, Ghanayem BI, Bell DA, Zhang ZY, Kaminsky LS, Shenfield GM (1996). The role of the CYP2C9-Leu359 allelic variant in the tolbutamide polymorphism. Pharmacogenetics.

[10] Nasu K, Kubota T, Ishizaki T (1997). Genetic analysis of CYP2C9 polymorphism in a Japanese population. Pharmacogenetics.

[11] Yang L, Ge W, Yu F, Zhu H (2010). Impact of VKORC1 gene polymorphism on interindividual and interethnic warfarin dosage requirement--a systematic review and meta analysis. Thromb Res.

[12] Rosemary J, Surendiran A, Rajan S, Shashindran CH, Adithan C (2006). Influence of the CYP2C9 AND CYP2C19 polymorphisms on phenytoin hydroxylation in healthy individuals from south India. Indian J Med Res.

[13] Aynacioglu AS, Brockmöller J, Bauer S, Sachse C, Güzelbey P, Ongen Z (1999). Frequency of cytochrome P450 CYP2C9 variants in a Turkish population and functional relevance for phenytoin. Br J Clin Pharmacol.

[14] Sconce EA, Khan TI, Wynne HA, Avery P, Monkhouse L, King BP (2005). The impact of CYP2C9 and VKORC1 genetic polymorphism and patient characteristics upon warfarin dose requirements: proposal for a new dosing regimen. Blood.

[15] Adithan C, Gerard N, Naveen AT, Koumaravelou K, Shashindran CH, Krishnamoorthy R (2003). Genotype and allele frequency of CYP2D6 in Tamilian population. Eur J Clin Pharmacol.

[16] Jose R, Chandrasekaran A, Sam SS, Gerard N, Chanolean S, Abraham BK (2005). CYP2C9 and CYP2C19 genetic polymorphisms: frequencies in the south Indian population. Fundam Clin Pharmacol.

[17] Rathore SS, Agarwal SK, Pande S, Mittal T, Mittal B (2010). Frequencies of VKORC1 -1639 G>A, CYP2C9*2 and CYP2C9*3 genetic variants in the Northern Indian population. Biosci Trends.

[18] Hashimoto Y, Otsuki Y, Odani A, Takano M, Hattori H, Furusho K (1996). Effect of CYP2C polymorphisms on the pharmacokinetics of phenytoin in Japanese patients with epilepsy. Biol Pharm Bull.

[19] Mamiya K, Ieiri I, Shimamoto J, Yukawa E, Imai J, Ninomiya H (1998). The effects of genetic polymorphisms of CYP2C9 and CYP2C19 on phenytoin metabolism in Japanese adult patients with epilepsy: studies in stereoselective hydroxylation and population pharmacokinetics. Epilepsia.

[20] Odani A, Hashimoto Y, Otsuki Y, Uwai Y, Hattori H, Furusho K (1997). Genetic polymorphism of the CYP2C subfamily and its effect on the pharmacokinetics of phenytoin in Japanese patients with epilepsy. Clin Pharmacol Ther.

[21] van der Weide J, Steijns LS, van Weelden MJ, de Haan K (2001). The effect of genetic polymorphism of cytochrome P450 CYP2C9 on phenytoin dose requirement. Pharmacogenetics.

[22] Lee A-Y, Kim M-J, Chey W-Y, Choi J, Kim B-G (2004). Genetic polymorphism of cytochrome P450 2C9 in diphenylhydantoin-induced cutaneous adverse drug reactions. Eur J Clin Pharmacol.

[23] Kesavan R, Narayan SK, Adithan C (2010). Influence of CYP2C9 and CYP2C19 genetic polymorphisms on phenytoin-induced neurological toxicity in Indian epileptic patients. Eur J Clin Pharmacol.

[24] Ozkaynakci A, Gulcebi MI, Ergeç D, Ulucan K, Uzan M, Ozkara C (2015). The effect of polymorphic metabolism enzymes on serum phenytoin level. Neurol Sci.

[25] Yamamoto Y, Takahashi Y, Imai K, Miyakawa K, Ikeda H, Ueda Y (2015). Individualized Phenytoin Therapy for Japanese Pediatric Patients With Epilepsy Based on CYP2C9 and CYP2C19 Genotypes. Ther Drug Monit.

[26] Yasar U, Eliasson E, Dahl ML, Johansson I, Ingelman-Sundberg M, Sjöqvist F (1999). Validation of methods for CYP2C9 genotyping: frequencies of mutant alleles in a Swedish population. Biochem Biophys Res Commun.

[27] Scordo MG, Caputi AP, D’Arrigo C, Fava G, Spina E (2004). Allele and genotype frequencies of CYP2C9, CYP2C19 and CYP2D6 in an Italian population. Pharmacol Res.

[28] Zand N, Tajik N, Moghaddam AS, Milanian I (2007). Genetic polymorphisms of cytochrome P450 enzymes 2C9 and 2C19 in a healthy Iranian population. Clin Exp Pharmacol Physiol.

[29] Azarpira N, Namazi S, Hendijani F, Banan M, Darai M (2010). Investigation of allele and genotype frequencies of CYP2C9, CYP2C19 and VKORC1 in Iran. Pharmacol Rep.

