# Supplementary material for: Frequencies of CYP2C9 polymorphisms in North Indian population and their association with drug levels in children on phenytoin monotherapy
Source: BMC Pediatr. 2016 May 14;16:66. doi: 10.1186/s12887-016-0603-0 (PMC4868001; doi:10.1186/s12887-016-0603-0)
Supplement: Additional file 1: — Polymerase chain reaction. PCR was performed by initial denaturation at 94 °C for 2 min followed by 35 cycles of denaturation at 94 °C for 30 s, annealing at 60 °C for 10 s and extension at 72 °C for 1 min followed by final extension at 72 °C for 7 min. The products were run at 250 V in a horizontal electrophoresis system (Bangalore Genie) on a 2 % agarose gel to check for amplification. 5 μL of PCR products (375 bp) were digested overnight at 37 °C with Ava II (New England Biolabs) for CYP2C9*2 genotyping. A 130 bp amplicon was digested by Sty 1 (Eco 1301) (Fermentas International Inc) for CYP2C9*3 genotyping. After the overnight digestion, the digested DNA was run along with the undigested PCR product on a 2 % agarose gel with ethidium bromide at 150 V in a horizontal electrophoresis system and visualized under UV light. (PDF 82 kb) [file 12887_2016_603_MOESM1_ESM.pdf]

Additional file 1

PCR was performed by initial denaturation at 94 ºC for 2 minutes followed by 35 cycles of denaturation at 94 ºC for 30 seconds, annealing at 60 ºC for 10 seconds and extension at 72ºC for 1 minute followed by final extension at 72 ºC for 7 minutes. The products were run at 250 volts in a horizontal electrophoresis system (Bangalore Genie) on a 2% agarose gel to check for amplification. 5 µL of PCR products (375 bp) were digested overnight at 37 ºC with *Ava II* (New England Biolabs) for CYP2C9*2 genotyping. A 130 bp amplicon was digested by Sty 1(*Eco 1301*) (Fermentas International Inc) for CYP2C9*3 genotyping. After the overnight digestion, the digested DNA was run along with the undigested PCR product on a 2% agarose gel with ethidium bromide at 150 volts in a horizontal electrophoresis system and visualized under UV light.
